# CRRT Is More Than Just Continuous Renal Replacement Therapy

**DOI:** 10.3390/ph17121571

**Published:** 2024-11-22

**Authors:** Lóránd Erdélyi, Domonkos Trásy

**Affiliations:** 1 Faculty of Helath and Sport Science, Széchenyi István University, 9026 Győr, Hungary; 2 Centre for Translational Medicine, Semmelweis University, 1085 Budapest, Hungary; trasydom@gmail.com

**Keywords:** continuous renal replacement therapy, CRRT, adsorption, pharmacokinetics

## Abstract

The physiology of the kidney has long been understood, and its mechanisms are well described. The pathology of renal failure is also a deeply researched area. It seems logical, therefore, to create devices that can replace the lost normal function of the kidney. Using the physical processes that take place in the kidney, such as diffusion or convection across a membrane, various renal replacement therapies (RRT) have been created. There are those that are used intermittently and those that are used for longer periods. What they have in common is that all RRTs have the same purpose; to replace the excretory function of the kidney that has been lost. CRRT is an extracorporeal renal replacement therapy that effectively replicates the excretory function of the kidneys in cases of acute renal failure. However, it has become increasingly evident that this rapidly advancing treatment modality offers benefits beyond merely substituting kidney function, with its applications continuing to expand significantly with non-renal and other indications. The use of these devices has raised new questions, many of which are still not clearly answered. When should this start? Who should receive it? How long should it last? What indication should it be for? What modality should it be with? How does it change the pharmacokinetics of the medicines? To answer these questions, it is first worth understanding the mechanisms behind the processes and the factors that influence them. This should not only focus on the procedures used in RRT therapies, but also consider the patient’s condition and the physicochemical properties of the drugs. In this review, we aim to provide a literature summary to highlight the factors that may influence the success of RRT therapies.

## 1. Introduction

The spread of continuous renal replacement therapy (CRRT) represents a significant milestone in critical care therapy, becoming a routine treatment in more and more intensive care units (ICUs). CRRT has joined the ranks of other vital organ support therapies, like invasive mechanical ventilation, circulatory support, hemodynamic monitoring, and fluid therapy. Constantly growing numbers of active inpatient facilities offer some form of extracorporeal organ support (ECOS), with CRRT being the most extensive.

There are still wide variations of CRRT rates and practices worldwide, and there are places where up to a fifth of all ICU patients receive CRRT treatment [1]. But it is important to recognize that clinicians often encounter numerous obstacles, including a shortage of nephrologists and trained personnel, gaps in knowledge, limited access to the necessary equipment, cultural and socioeconomic challenges, high therapy costs without reimbursement, and both administrative and governmental hurdles, on a global scale.

CRRT is an extra-corporal renal replacement procedure that can highly effectively replace the excretory function of an insufficiently functioning kidney in acute renal failure [2]. However, it is increasingly clear that this rapidly evolving therapeutic modality extends beyond just replacing kidney function, with its indications expanding rapidly.

In many settings, CRRT has replaced previously established intermittent treatments. Although no randomized controlled trial (RCT) has definitively proven the superiority of CRRT, various factors affecting mortality have shown better outcomes with CRRT. Thus, the current KDIGO (Kidney Disease Improving Global Outcomes) guidelines recommend CRRT as the first line of therapy (2B) in cases of acute kidney injury (AKI) accompanied by hemodynamic instability [3].

The use of these devices has raised new questions, many of which are still not clearly answered. When should this start? Who should receive it? How long should it last for? What indication should it be provided for? What modality should it be with? How does it change the pharmacokinetics of the medicines? To answer these questions, it is worth first understanding the mechanisms behind the processes and the factors that influence them. This should not only focus on the procedures used in RRT therapies but should also take into account the patient’s condition and the physicochemical properties of the drugs. In this review, we aim to provide a literature summary to highlight the points that may influence the success of RRT therapies.

## 2. Kidney Physiology and the Relationship Between RRTs

The function of the kidneys, and therefore the formation of urine, can be divided into the following three processes: glomerular filtration, reabsorption, and secretion.

Glomerular filtration takes place in the Bowman’s capsule, and occurs due to hydrostatic pressure difference. Because of the lower pressure inside the Bowman capsule, the fluid and its solutes are pushed into the Bowman capsule.

The membrane that forms the filtrate consists of three layers. The first layer is the fenestrated endothelium of the glomerular capillaries, which is permeable to all blood components except cells. The second layer is the basement membrane, which is a negatively charged membrane that have the function of preventing the permeation of proteins. The third layer is made up of podocytes, which allows for even more selective filtration.

The formed filtrate undergoes reabsorption processes. The four different tubular sections reabsorb the substances the body needs by a specific mechanism. The four segments are the proximal convoluted tubule, the nephron loop, the distal convoluted tubule, and the collecting tubule. This is where glucose, amino acids, ions, vitamins, and water are reabsorbed [4].

In addition to tubular reabsorption, tubular secretion processes are also present. This is where many small molecules, that may include antibiotics, antivirals, diuretics, NSAIDs, metabolites, and toxins are secreted [5].

Kidney failure can result from the malfunctioning of the mechanisms mentioned above. The RRT procedures used in the treatment of renal failure replace these mechanisms for shorter or longer periods of time.

RRT processes are based on two mechanisms: diffusion and convection. In both mechanisms, the solution containing the blood and the excreted material must be separated by a semipermeable membrane. The process seems simple; however, several variables increase its complexity. In all cases, the blood flow rate, dialysate flow rate and concentrations must be considered. In addition, convection increases the size range of molecules that can pass through the membrane, and the varying specifications of the membrane material and surface area can greatly alter the efficiency of the process [6].

Depending on the clinical condition of the patient and the desired therapeutic goal, different RRT procedures can be chosen. Intermittent or continuous procedures may be used. The two procedures have important differences that should be taken into account when planning the therapy.

## 3. IHD, CRRT Differences

For greater clarity, it is worth considering the differences between intermittent hemodialysis (IHD) and continuous renal replacement therapy (CRRT) (see Table 1).

### 3.1. Mechanism

During IHD, the process that takes place is diffusion only. In contrast, in CRRT treatment, depending on the modality used, it may be diffusion, convection or both. The mechanisms can greatly affect the removal of drugs (see later for a description of the mechanisms).

### 3.2. Flow Rates

Both blood and dialysate flow rates are higher during IHD. While IHD operates at >200–300 mL/min blood flow rate and >500 mL/min dialysate flow rate, CRRT operates at <200 mL/min blood flow rate and 17–34 mL/min dialysate flow rate.

### 3.3. Duration

In terms of duration, IHD, as the name suggests, is shorter than CRRT. IHD can be applied every day or several days a week depending on the patient’s condition, but on average a single treatment lasts 3–4 h. CRRT, on the other hand, can run continuously for days if there are no technical problems.

### 3.4. Advantages—Disadvantages

Due to the higher flow rates of IHD, compared to CRRT, it has more rapid fluid removal and a more rapid clearance. However, it may induce hemodynamic instability to a greater extent than CRRT. This is why CRRT is a gentler procedure for hemodynamically unstable patients. In addition, CRRT, when used in a hemofiltration or hemodiafiltration modality, is able to remove medium molecular weight substances. However, there is one more very important issue to consider, and that is the cost. The CRRT procedure is significantly more expensive than IHD treatment, not only in financial terms but also in terms of human resources [7].

## 4. Application of CRRT at Bedside

### 4.1. CRRT Indications

The indications for CRRT are primarily determined by the patient’s current therapeutic needs, considering their clinical response to initial treatments. The main objective is to temporarily replace or support failing organ functions, allowing the body time to heal and regenerate. This is true even if the primary organ systems are not yet fully failing but are expected to deteriorate based on the anticipated pathophysiological course. Additionally, if the disease process is likely to place increased demands on other organs, leading to secondary organ damage and potentially multiple organ failure, the goal of therapy may include reducing the burden on these systems, thereby preventing further organ damage, even if that is not yet clinically apparent. CRRT may also be indicated for bridging until definitive treatment of irreversible organ damage can be provided.

A new perspective is emerging that places significant emphasis on prevention alongside addressing current therapeutic needs. Experimental and clinical research suggests that early extracorporeal organ support may effectively prevent dysfunction and failure in organs that are not yet damaged, with most cases showing a significant improvement in organ function as a result of the therapy.

Indications for CRRT can be broadly categorized into three main areas, though they often overlap and are not mutually exclusive.

#### 4.1.1. Renal Indications

Among the classical indications, the most well-known and significant is the therapeutic need arising from acute kidney injury (AKI). It is crucial to emphasize that renal replacement therapies (RRT) do not address the underlying cause of renal failure but are supportive measures for maintaining vital functions, whether in cases of acute or chronic kidney failure. The primary issue is not only the accumulation of urea and creatinine, which are typically monitored to assess kidney function, but also the life-threatening conditions resulting from dysregulated processes, such as hyperkalemia (potassium levels > 5.6 mmol/L), metabolic acidosis (pH < 7.1), and hypervolemia. These conditions, though often associated with kidney failure, can arise independently, and in such cases, CRRT aims to remove toxins that are not cleared by the kidneys, restore acid–base balance, manage fluid balance, and correct electrolyte imbalances. Other indications include complications of kidney failure, such as uremic encephalopathy, pericarditis, or bleeding tendencies.

While we have reliable biochemical methods to monitor kidney function, allowing us to track the onset, progression, and severity of AKI, the exact timing for initiating CRRT remains uncertain, a topic addressed in the following section.

**Figure 1:**
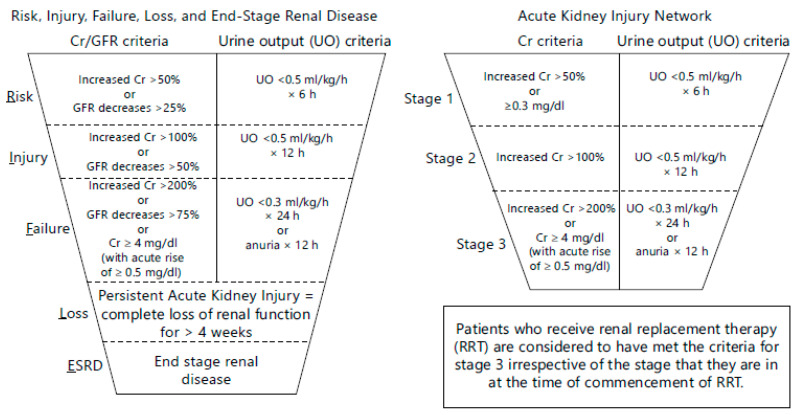
The RIFLE and AKIN classification and staging of acute kidney injury [9].

AKI is defined as a reduction in kidney function, including decreased glomerular filtration rate (GFR). The criteria for diagnosing AKI and determining its severity are based on changes in serum creatinine (SeCr) and urine output. In 2004, the RIFLE (Risk, Injury, Failure, Loss of kidney function, and End-stage kidney disease) classification was introduced to define and stratify the severity of acute kidney injury [8]. This system relies on changes in SeCr or GFR and/or urine output and has proven effective in identifying AKI patients across various settings, monitoring AKI severity, and predicting patient outcomes. Three years later, in 2007, the Acute Kidney Injury Network (AKIN) classification, a modified version of RIFLE, was introduced to improve the sensitivity and specificity of AKI diagnosis [9,10] (see Figure 1).

**Figure 2:**
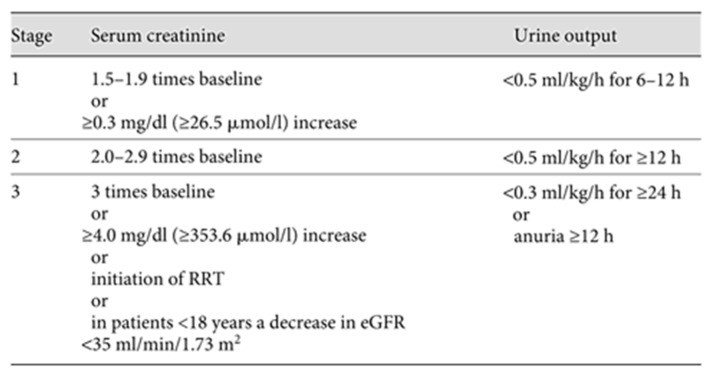
Proposed KDIGO staging of AKI.

In 2012, KDIGO published an updated guideline, which remains relevant today, offering a comprehensive overview of AKI management from prevention to prognosis and treatment. KDIGO classifies AKI into three stages (KDIGO stages one, two, and three), primarily based on mortality risk and the need for RRT [3] (see Figure 2).

#### 4.1.2. Non-Renal Indications

Diagnosis-based or non-renal indications refer to the need for CRRT due to organ failures or pathologies other than kidney function. These indications often involve the removal of larger molecules as a symptomatic treatment, which has recently become the primary indication for CRRT, surpassing AKI. The preventive approach is increasingly emphasized in these cases.

These indications include conditions such as severe infections (e.g., sepsis, acute respiratory distress syndrome (ARDS), severe peritonitis, or deep soft tissue infections). Even in cases of sterile conditions, such as severe pancreatitis, the removal of middle-size molecules (cytokines, inflammatory mediators) may be beneficial to stabilize organ function.

CRRT may also be indicated for cardiac reasons, particularly in cases of cardiogenic shock or congestive heart failure. It can also aid in stabilizing patients in the early postoperative phase following cardiac surgery.

In conditions associated with severe tissue damage, such as extensive surgery, major trauma, burns, or prolonged ischemia-reperfusion injury, the breakdown products can activate damage-associated molecular patterns (DAMPs), leading to severe organ failure, including rhabdomyolysis. Similarly, tumor lysis syndrome can create a comparable scenario, where CRRT may provide crucial support.

Massive fluid overload (body weight > 10–20%), leading to refractory hypervolemia despite pharmacological treatment, can worsen cardiac and respiratory status, and cause abdominal compartment syndrome, ascites, pleural effusions, and even symptomatic cerebral edema, all of which can be effectively managed with CRRT. It is also important to mention conditions following massive transfusion, where, in addition to volume overload, hyperkalemia, hypocalcemia, and transfusion-related acute lung injury (TRALI) can lead to ARDS.

Accidental or intentional drug overdoses and other toxicities can also be treated with CRRT. Additionally, CRRT may be beneficial in immunological, neurological (e.g., Guillain–Barré syndrome), hematological (e.g., immune thrombocytopenia, thrombotic thrombocytopenic purpura), or HELLP syndrome cases.

Other indications include liver failure, hyperlipidemia, hyperviscosity disorders, or neurological conditions such as prolonged convulsions leading to metabolic and electrolyte imbalances, severe delirium tremens, or neuroleptic malignant syndrome. These non-evidence-based, empirical indications are diverse, and the list could go on.

These indications are not based on strong evidence supported by studies; there are no statistics on how different patient groups respond, and the effectiveness of the therapy is difficult to assess objectively. Instead, clinicians’ decisions in this regard are primarily guided by daily practice, experience, and the intention to heal. Therefore, objective research results are urgently needed to gain a more accurate understanding of these indications.

#### 4.1.3. Indications Based on Response to Initial Therapy

This section primarily concerns the immediate correction of non-specific life-threatening conditions with dialysis treatment, which, in the short term, could potentially lead to the patient’s death, regardless of the underlying causes.

In the case of acid–base disturbances, this might include the rapid and effective correction of severe or prolonged metabolic acidosis with dialysis, or simply preventing the condition from worsening. Additionally, in cases of elevated lactate levels or a persistent need for high catecholamine support, initiating CRRT can provide rapid symptomatic relief.

In cases of severe, life-threatening thermoregulatory disorders, such as hypothermia or hyperthermia, the most effective and fastest treatment method is intravascular temperature control. By directly cooling or warming the blood, we can affect the body’s core temperature, indirectly reaching all tissues and cells without directly damaging them. It should be noted that under certain circumstances, the goal is not speed, but a controlled, gradual change in temperature.

For severe, life-threatening electrolyte imbalances, CRRT is also one of the fastest and most effective treatment methods. It can be lifesaving, especially in cases of hyperkalemia, although when dealing with severe sodium imbalances, care must be taken to follow the principle of gradual correction to avoid secondary severe complications. In such cases, non-standard, specially formulated electrolyte solutions may be needed.

Another important life-threatening condition is hypervolemia, which can also be quickly and controllably managed with continuous dialysis treatment. In clinical practice, this problem is most often encountered in congestive heart failure, but it can be lifesaving in cases of pulmonary edema, ARDS (with high extravascular lung water), and even high intraabdominal pressure or symptomatic cerebral edema.

It is important to emphasize that the line between the different indications is often blurred, and several reasons can simultaneously justify the initiation of CRRT. While guidelines generally suggest that CRRT should be initiated when life-threatening conditions cannot be corrected pharmacologically, the rapidly expanding list of CRRT indications shows that its therapeutic potential is vast and, while currently underutilized, offers significant possibilities.

### 4.2. Timing of CRRT Initiation

Currently, there is ongoing active research into the optimal timing for starting continuous renal replacement therapy. However, no definitive conclusions have been reached so far. The critical care patient population, including those with sepsis, is difficult to standardize, and there is limited evidence available.

The most crucial focus should be on prevention. This includes discontinuing nephrotoxic agents, optimizing volume status and perfusion pressure, conducting hemodynamic monitoring, avoiding hyperglycemia and radiocontrast materials, and closely monitoring drug dosages. In most cases, acute kidney injury arises from prerenal causes, primarily due to hypovolemia or sepsis [11]. Renal and postrenal causes follow in frequency.

If dialysis looks unavoidable, as with any treatment, it should be individualized, considering the patient’s circumstances and needs. Factors such as the patient’s current clinical condition, the rate of deterioration, age, underlying and comorbid conditions, and future treatment plans (e.g., anticipated surgical interventions) are important.

In cases of acute kidney failure, the baseline condition is crucial, as many individuals already live with compromised kidney function. The diagnostic criteria previously detailed (RIFLE, AKIN, and KDIGO) do not provide clear guidance on when to start therapy. Some initiate treatment at the risk stage, while others wait until the failure stage or stage three. This is because creatinine changes are typically delayed and are not reliable indicators of when to start RRT. Their value is influenced by many factors, including non-renal factors and hydration status. Early biomarkers, like troponin-like proteins, NGAL (Neutrophil Gelatinase-Associated Lipocalin) [12], or Cystatin C [13] have not proven to be sufficiently reliable, and thus are not yet included in any diagnostic criteria.

In recent years, numerous studies have investigated the optimal timing for initiating dialysis therapy due to renal indications, yet the results remain contradictory. These studies have been highly heterogeneous regarding patient populations, sample sizes, disease severity, treatment modalities and protocols, endpoints analyzed [14,15,16,17,18]. The studies’ common thread is their focus on comparing the effects of early-versus-delayed dialysis initiation on organ function, outcomes, and survival.

However, while no definitive conclusions could be drawn from these studies, a tentative consensus appears to suggest that the optimal treatment approach for classic renal indications seems to be neither too early (first day) nor too late (after the third day). This relatively broad interval is determined based on survival outcomes.

In practice, clinicians often delay RRT initiation if they believe that the patient has a good chance of recovering without it, considering the potential complications of treatment (e.g., hemodynamic instability, arrhythmias, membrane biocompatibility, dialysis and catheter issues, and anticoagulation complications). According to current knowledge, it is recommended to initiate treatment within the 24–72 h window after AKI diagnosis, as numerous studies confirm the positive impact of timely treatment on outcomes and kidney function recovery.

Even less data are available for non-renal indications. Individual judgment is needed to determine the appropriate indications and timing of therapy based on the patient’s needs.

In cases where therapy is initiated based on response indications, the timing is less flexible, as these are often urgent, life-threatening conditions where the patient’s survival may depend on timely, effective dialysis treatment.

### 4.3. Termination of Therapy

A critical aspect of renal replacement therapy is determining when the patient no longer requires treatment. Unfortunately, in some cases, acute kidney injury (AKI) can progress to end-stage renal failure, leading to chronic dialysis. The likelihood of this progression increases over time, with end-stage renal disease often being confirmed around the third month if organ function remains impaired.

In cases of renal indication, treatment can be considered for cessation when the patient has resumed spontaneous diuresis (>400–500 mL/day) and, despite dose reduction, the patient remains metabolically stable, has good creatinine clearance, and maintains balanced electrolytes. Initially, during continuous renal replacement therapy (CRRT), a treatment holiday is often taken during circuit changes. If stopping the treatment does not succeed, and metabolic, electrolyte, and fluid balance cannot be maintained, or if cardiovascular instability occurs, new CRRT sessions will be initiated. For stable patients, intermittent renal replacement therapy may be continued.

In critically ill patients, even with improving conditions, therapy may continue, focusing on removing excess edema and managing fluid status in the final days. This helps to restore metabolic balance and facilitates cellular and tissue regeneration.

For therapies initiated for non-renal indications, the duration may be pre-determined or based on achieving specific therapeutic goals. In critically ill patients, the primary goal is often the stabilization of organ functions. CRRT may be discontinued when organ functions, including renal, hemodynamic, and gas exchange functions, are normalized.

In cases where treatment is initiated based on a response indication, the primary objective is to resolve acute life-threatening conditions. However, due to potential additional organ failures or complications, treatment may continue until full vital stability is achieved.

## 5. Processes Influencing Pharmacokinetics During CRRT Treatment

### 5.1. Operating Principle—Basic Laws

In order to understand the functioning of CRRT and the mechanisms that modify its pharmacokinetics, it is essential to know the applicable modalities and principles. Different modes have different driving forces that affect fluid transportation and thus the excretion of substances dissolved in the blood, including medicines.

We look at three modes of CRRT, veno-veno hemodialysis (CVVHD), veno-veno hemofiltration (VVVH) and veno-veno hemodiafiltration. (CVVHDF). In hemodialysis, diffusion is the process played and in the hemofiltration process, convection is the process played, while in hemodiafiltration, the two processes occur simultaneously [19].

In addition to diffusion and convection mechanisms, adsorption can also take place on the surface of a CRRT membrane. Convection and diffusion are also present during the physiological functioning of the kidneys, so first the primary filter is formed, and then the urine. Adsorption, meaning binding on the surface, is not a typical process in the kidneys, since there is no tissue capable of adsorbing substances dissolved in water [20]. The three processes are driven by different forces.

#### 5.1.1. Diffusion

**Figure 3:**
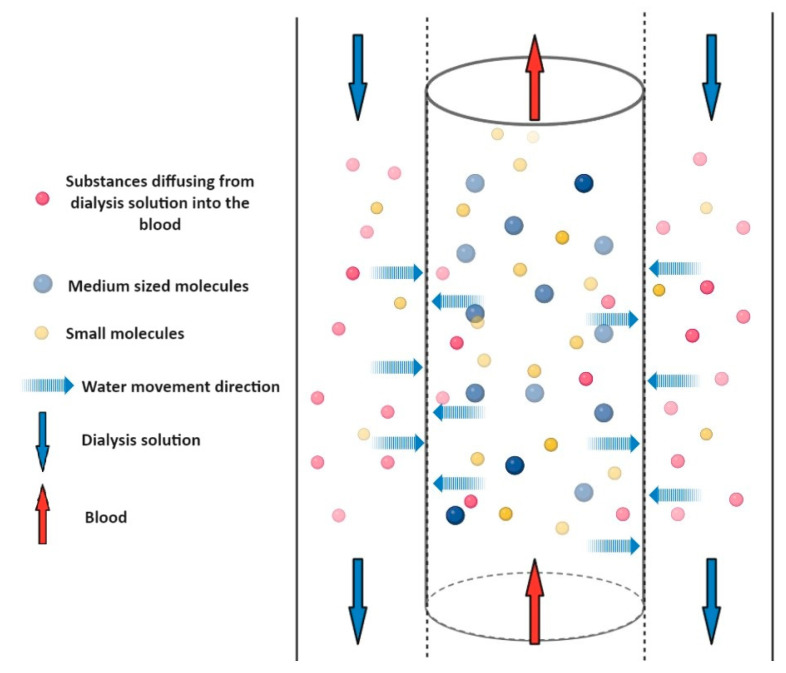
Schematic representation of diffusion. A semi-permeable membrane separates the blood in the central cylinder from the dialysis fluid marked in the side columns. The two liquids flow in opposite directions (marked by the red and blue arrows). The blue dashed arrows show that water and solutes can pass through the membrane in both directions. The direction of crossing is determined by the concentration gradient. The size of the molecule greatly influences the possibility of crossing the membrane. Medium molecules (blue dots) cannot cross the membrane, and therefore remain in the blood and cannot be dialyzed. Small molecules in the blood (yellow dots) are able to pass through the membrane and small molecules in the dialyzing solution (pink dots) are able to enter the blood.

Diffusion is described by Fick’s laws, and means the movement and distribution of the solvent and the dissolved substance, depending on the concentration and finally the equalization of the concentrations. (see Figure 3) In this transport process, the concentration gradient is the driving force of the process, so if the concentration equalizes between the two sides of the membrane, the process seems to stop. This process can occur simultaneously through the membrane in both directions. If the concentration of a specific ion, such as bicarbonate, in the dialysis solution is higher than in the blood, then it diffuses in the direction of the blood. For example, passive reabsorption of water in the kidney takes place with this mechanism. In the proximal tubule or descending branch of the loop of Henle, water passively diffuses into the blood vessels thanks to the suction force of the blood with a high osmotic pressure circulating there. Low molecular weight (<500–1000 Da) drugs can generally be removed by diffusion (hemodialysis), because they pass through the pore size of the membrane and are transported with water as their solvent.

#### 5.1.2. Convection

**Figure 4:**
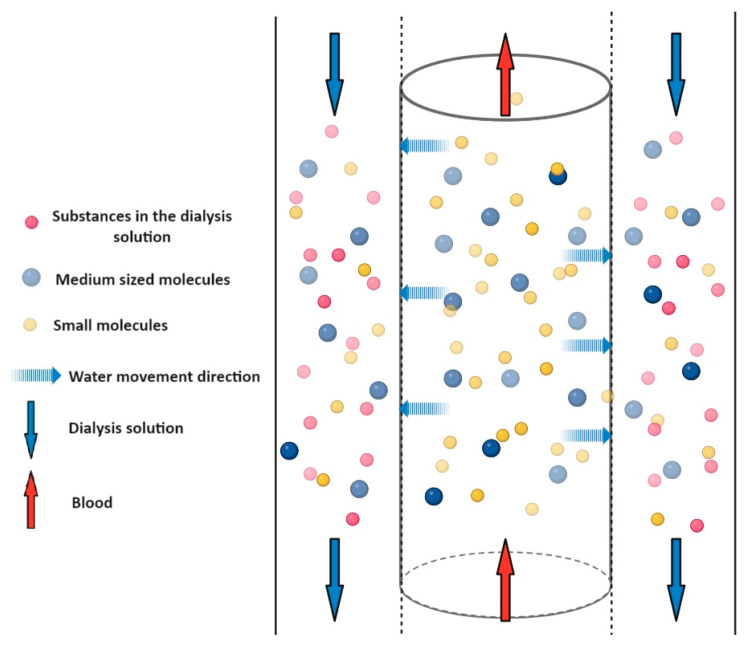
Schematic representation of convection. A semipermeable membrane separates the blood in the central cylinder from the solution marked in the outer columns. The two fluids flow in opposite directions (red and blue arrows). The blue dashed arrows show that water and solutes from the blood can pass to the other side of the membrane. The direction of crossing is determined by the hydrostatic pressure difference. Medium molecules (blue dots) can also cross the membrane and are therefore present on both sides of the membrane. Small molecules in the blood (yellow dots) can also pass out of the blood through the membrane, but no substances enter the blood (pink dots are not on the blood side).

During convection (involving mass transport and bulk flow), the solute flows together with the solvent (see Figure 4). Convection is caused by the pressure difference through a porous membrane, whether that is a dialysis membrane or the vascular wall. The solvent and solute flow from the higher pressure to the lower pressure site. Since the pressure difference is the driving force in the process, no dialysis solution is needed. It does not need to provide suction power based on the concentration difference. In this process, the pore size of the membrane is a determining factor in terms of the passage of molecules. Medium molecular weight substances can already be removed with this mechanism, and filtrate is formed in the Bowman’s capsule located in the kidneys with this mechanism. The increased blood pressure (about 50 mmHg) in the arteriole is due to the fact that the diameter of the exit arteriole is smaller than that of the entrance.

#### 5.1.3. Adsorption

**Figure 5:**
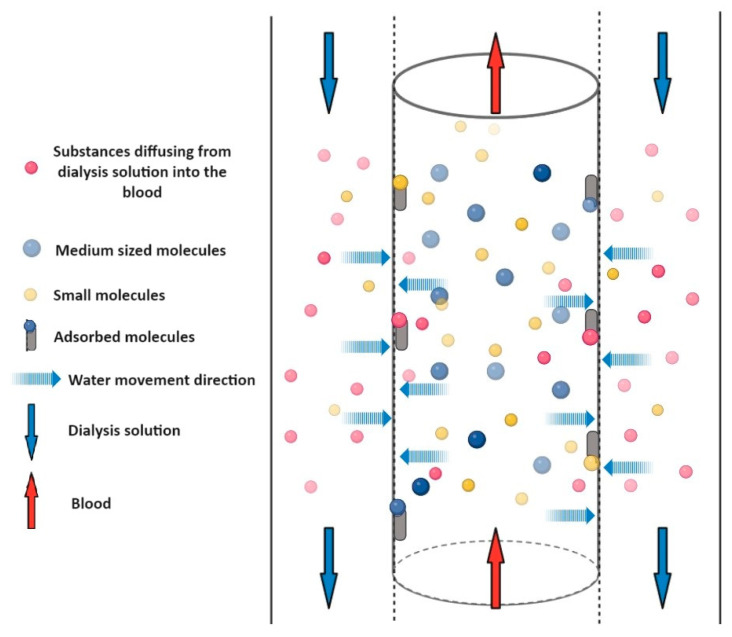
Schematic representation of adsorption. A semipermeable membrane separates the blood in the central cylinder from the solution marked in the outer columns. The two liquids flow in opposite directions (red and blue arrows). The blue dashed arrows show that water and solutes can pass through the membrane in both directions, so in this case, we are talking about diffusion. The direction of crossing is determined by the difference in concentration gradient. The medium molecules (blue dot) cannot cross the membrane and are therefore not present on either side of the membrane. Small molecules in the blood (yellow dots) can also exit the blood through the membrane and substances can enter the blood (pink dots). Substances bound on the membrane surface (grey slits with colored dots) show possible adsorption.

Adsorption means binding on the surface (see Figure 5), and this process can be reversible or irreversible from the point of view of binding to the surface. Binding is influenced by the material quality of the membrane used, the physico-chemical properties of the materials that interact with the membrane, and their concentration. Depending on these, second-order chemical bonds of different strengths can be formed [21]. In some cases, the process of adsorption can be the basis of the procedure. In the treatment of poisoning caused by a toxin or drug, an adsorbent with a designed high-adsorption capacity can quickly and efficiently remove the agent causing the poisoning. At the same time, in the case of a dialysis membrane, this can appear as an unwanted process. Although dialysis membranes have a much lower adsorption capacity, they are able to bind active substances to varying degrees, thus influencing their plasma concentration.

#### 5.1.4. Pre- and Post-Dilution

**Figure 6:**
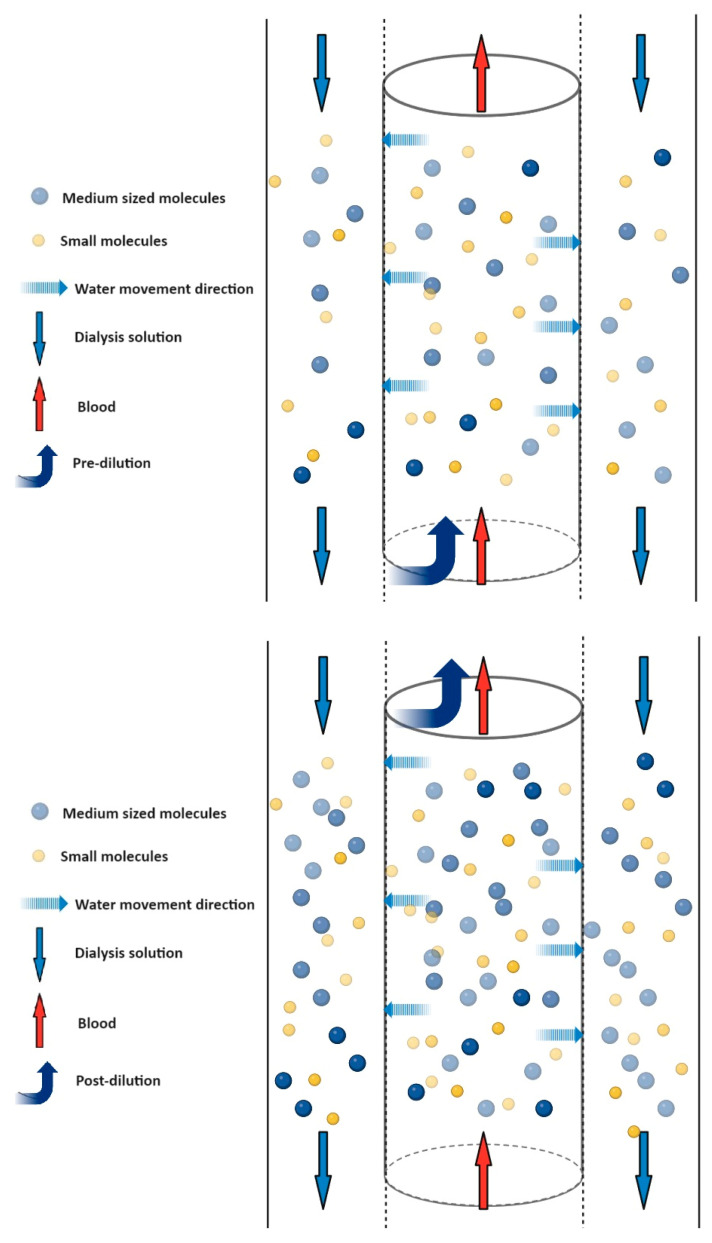
Higher material concentration with post-dilution. A semipermeable membrane separates the blood in the central cylinder from the solution marked in the outer columns. The two liquids flow in opposite directions (red and blue arrows). The blue dashed arrows show that water and solutes only flow outwards through the membrane, so that convection takes place. The direction of crossing is determined by the pressure difference. Medium molecules (blue dot) can also pass through the membrane and are therefore present on both sides of the membrane. Small molecules (yellow dots) in the blood also exit the blood through the membrane. When using pre- or post-dilution, we can observe a difference in concentrations. As shown in the figure, when using pre-dilution, the concentration of the substances (blue and yellow dots) is reduced compared to the post-dilution procedure, due to the extra solution added.

The measure of the mechanisms mentioned above is clearance, which means the amount of plasma that the kidney or kidney replacement procedure completely clears from a given substance in a given time. The effectiveness of this is significantly influenced by whether the kidney replacement procedure is performed pre- or post-dilution. In the case of pre-dilution, the substitution fluid is added to the extracorporeal circuit before the membrane, while in post-dilution, it is added after the membrane (see Figure 6). Both procedures have advantages and disadvantages. In addition to post-dilution, for example, a disadvantage is the increased blood clotting tendency, but there is a higher filtration efficiency; in pre-dilution, the opposite is true. The these differences can be explained by the concentration of the substances in the blood that meet the membrane. In the case of pre-dilution, the ions, metabolites, and drugs in the blood reach the dialysis membrane at a lower concentration, so their removal is less efficient.

### 5.2. Determination of CRRT Clearance

It is also possible to describe clearances related to different modalities using equations. Based on this, we can say that neither procedure takes into account the process of adsorption. The filtration coefficient and saturation coefficient or the ultrafiltrate and blood flow rate are identified as the determining parameters.

Equations:

$${Cl}_{x}=C_{V(mM)}\times \frac{V_{\left(mL/min\right)}}{C_{P(mM)}}$$

$${Cl}_{CVVH(post)}=Q_{f}\times Sc$$

$${Cl}_{CVVH(pre)}=Q_{f}\times Sc\times \frac{Q_{b}}{Q_{b}+Q_{rep}}$$

$${Cl}_{CVVHD}=Q_{d}\times S_{d}$$

$${Cl}_{CVVHDF}=\left(Q_{f}+Q_{d}\right)\times S_{d}$$

where ${Cl}_{X}$: selected (x) substance clearance; $C_{V}$: urine concentration of excreted substance (x); V: amount of urine excreted per unit time (minutes, seconds); $C_{P}:$ plasma concentration of excreted substance (x); ${Cl}_{\mathrm{CVVH}(\mathrm{post})}$, ${Cl}_{\mathrm{CVVH}(\mathrm{pre})}$: clearance in CVVH modulation in post- or pre-dilution; ${Cl}_{\mathrm{CVVHD}}$: clearance when CVVHD is used; ${Cl}_{\mathrm{CVVHDF}}$: clearance when CVVHDF is used. The variables were the following: $Q_{f}$: ultrafiltrate flow rate; $Q_{b}$: blood flow rate; $Q_{\mathrm{rep}}$: substitution fluid flow rate; $Q_{d}$: dialysate flow rate; $S_{c}$: sieving coefficient; $S_{d}$: saturation coefficient [22].

The first equation is a general description of renal clearance, which focuses on urine and plasma concentrations, neglecting the mechanisms involved. In contrast, when describing CRRT clearance values, it can be seen that there are variables that can be used to influence the degree of clearance.

The filtration coefficient and saturation coefficient indicate the degree to which a given substance is capable of filtration through the membrane. The filtration coefficient is used to describe hemofiltration, and the saturation coefficient is used to describe hemodialysis. If their value is one, the test substance can pass completely through the membrane, so concentration equivalence can be achieved even without external influences. If the value is less than one, the membrane is more difficult to permeate for the given material, so the concentrations between the donor and recipient side are not balanced without external influence. A value greater than one can only be achieved by using external energy [10].

In the equation describing the clearance of the CVVH procedure applied by pre-dilution, we can see that a correction factor appears. This is intended to correct the decrease in the concentration of dissolved substances in the blood due to pre-membrane dilution. The correction factor depends on the blood flow rate and the rate of addition of substitution fluid. The value is always less than one, so the pre-dilution clearance will always be less than the post-dilution clearance value [23].

### 5.3. Possible Pharmacokinetic Changes

The excretion of a drug molecule from the body is influenced by several factors. The main characteristics that significantly determine the pharmacokinetics of a drug are the apparent volume of distribution (VD), protein binding, and molecular size. Apart from the properties of the active substance, the membrane used in the extracorporeal procedure must also be taken into account. Such parameters are the size of the membrane surface, the filtration and saturation coefficient, and the material of the membrane.

The apparent volume of distribution (VD) shows how well the drug used remains in the intravascular space. If the VD is low (<1 L/kg), the active substance typically exits the intravascular space slightly, but if the value is high (>5 L/kg), the tissue distribution is beneficial. Thus, the partition coefficient significantly influences the concentrations that are measured in the blood, and therefore also the extent to which it can be removed from the body. In general, it can be said that the pharmacokinetics of drugs with a high VD value are probably not significantly changed by extracorporeal renal replacement therapy. On the other hand, drugs with a low VD value are more easily accessible from the bloodstream, so their excretion can be expected to a greater extent [24].

Drugs introduced into the body bind to proteins to varying degrees. From the pharmacological point of view, the active fraction can be considered the free fraction that is not bound to the protein. Active substances that show a high degree of protein binding achieve lower free active substance concentrations in plasma. By binding to the protein, the active substance reaches such a large size range that it will no longer be able to pass through the membrane, so the protein-bound fraction of the active substance cannot be removed by dialysis.

There are large molecules in themselves that, even in the free fraction that is not bound to the protein, fall into the size range in which passing through the membrane is not favored. These active substances or toxins cannot be removed by dialysis, so their pharmacokinetics do not change significantly during a CRRT treatment. However, during the kidney replacement treatment, there is also a process that cannot take place during the normal functioning of the kidney. As already mentioned, the kidney does not have the necessary surface for adsorption, so this process has no effect on the quantitative and qualitative composition of urine. However, an interaction can occur between the surface of the polymer of the dialysis membrane and the material present. The resulting bond can be the dispersion bond, ionic bond (electrostatic attraction force), hydrogen bond, or van der Waals forces. This raises the question as to what extent this process can influence the pharmacokinetics of drugs used during renal replacement therapy. The appearance and degree of adsorption is influenced by the material quality of the membrane and the size of its surface.

In terms of material quality, a cardinal question is whether there is any functional group with a charge present on the surface of the membrane-building polymer. If the membrane has a charge, it is more likely that an electrostatic attraction force will develop with charged molecules. The first membrane used was AN69, a membrane containing polyacrylonitrile (PAN) and anionic sulfonate groups. Currently, the most commonly used membranes are made of polysulfone (PS), polyamide (PA), polymethylmethacrylate (PMMA), and polyethersulfone (PES) polymers [25].

There have been relatively few studies examining the adsorption properties of membranes for drugs. The limited available literature almost exclusively examines the binding of antimicrobial drugs. The issue of interactions between proteins and membranes, however, is already a deeply investigated area. This is also due to the fact that their interaction significantly affects biocompatibility, such as membrane-induced blood clotting. The degree of charge present on the membrane surface can be quantified by determining the membrane zeta potential. From this value, it is possible to deduce the degree of expected adsorption [26,27]. In absolute terms, the higher the zeta potential of a membrane, the more likely it is that some interaction will occur with oppositely charged molecules.

Some in vitro studies show that the membrane used in the CRRT procedure can also influence the plasma concentration of drugs by adsorption. AN69ST (polyacrylonitrile membrane with polyethyleneimine coating) and PS (polysulfone) membranes were compared in the trials, by measuring the decrease in the concentration of vancomycin, gentamycin, ciprofloxacin, and tigecycline. They concluded that, especially for the AN69ST membrane, some degree of adsorption of the active substance may be clinically relevant. Due to the structure of the PS membrane, it binds to the tested antibiotics to a lesser extent [28,29]. However, adsorption is definitely worth bearing in mind as a possible mechanism. The concentration of the active substance may be modified, and thus the outcome of therapy may be influenced.

## 6. Properties of Drugs That Affect Pharmacokinetics

### 6.1. Absorption

Drug absorption in critically ill patients can be affected by a variety of factors. For example, during treatment with a vasopressor, due to reduced microcirculation (intestinal microvilli, skin), enteric or subcutaneous absorption may be reduced [30]. In critically ill patients, vomiting and diarrhea may occur. This may modify transit time, similar to drugs that may have such side effects. This may also alter metabolic activity, and pH conditions may change. Therefore, in a critically ill patient, parenteral drug administration is advisable [31]. If the amount of drug absorbed is also in question, it is almost impossible to adapt it to CRRT treatment. For this reason, it is better to administer the medicine parenterally.

### 6.2. Volume of Distribution

The volume of distribution shows how a medicine is distributed in the body. Drugs with a high volume of distribution can easily leave the bloodstream and penetrate surrounding tissues. Once the drug leaves the bloodstream and is able to accumulate in the tissues, removal becomes less effective with CRRT. For critical patients, the actual volume of distribution may differ greatly from the estimated volume of distribution. A severe hemorrhage, ascites, or oedema can alter the body’s water spaces to such an extent that it can affect the volume distribution [32].

### 6.3. Molecular Weight and Protein Binding

The size of the drug molecule strongly influences the removability of the drug by CRRT. The pore sizes of the membranes used in CRRT range from 20,000 to 300,000 daltons [33]. Depending on the modality used, molecules of different sizes can pass through the membrane at different rates. In the case of diffusion, the diffusion rate is inversely proportional to the molecular size, and the diffusion rate decreases rapidly for molecules larger than 500 daltons. Most drug molecules are smaller than 500 daltons. Thus, they can usually be removed by hemodialysis [32].

CRRT removes only the free, non-albumin-bound drug fraction. Therefore, the degree of protein binding specific to the drug is a significant factor. If a drug is more than 90% bound to plasma protein, its removability is reduced. This is explained by the fact that albumin alone is already 68,000 daltons in size. Therefore, even albumin that is not bound to a drug cannot cross the membrane because of its size, and the drug that is bound to it increases its size even further.

With reduced serum albumin, we would expect lower serum protein binding, and hence an increased free drug fraction. This increase is not always significant, however, because as the free drug fraction increases, the distribution also changes. A new balance will be established between tissues and blood, so that the decrease in serum protein will be partially compensated.

### 6.4. Permeability

Another important physico-chemical property of medicines is their solubility, which can be termed hydrophilic and lipophilic. Hydrophilic drugs cannot passively pass across cell membranes. Accordingly, they are distributed in plasma and extracellular space. They are also usually excreted via the kidneys. However, lipophilic drugs are able to cross cell membranes. Accordingly, their distribution will be different from the hydrophilic drugs, appearing in the intracellular space. Their excretion must be preceded by a metabolic process [34].

### 6.5. Importance of Inactive Ingredients

Solubility is also an important factor in the formulation of medicine. For example, voriconazole, due to its limited water solubility, should be formulated for IV use using sulfobutyl-ether-beta-cyclodextrin sodium (SBECD). This vehicle cannot be excreted via the kidney in cases of impaired renal function. If creatinine clearance is <50 mL/min, parenteral formulation is contraindicated. However, CRRT treatment, in the CVVH modality, can influence the pharmacokinetics of SBECD to such an extent that IV can be used, whereas this is not possible with intermittent hemodialysis. In a non-intermittent renal replacement procedure, SBECD exposure increases 6–7-fold compared to patients with normal renal function [35]. This example shows that, in addition to the active substance, sometimes vehicles must also be considered in therapy.

## 7. Therapeutic Modifications

As an example, we consider some cases where the characteristics of the RRT procedure affect the use of medicines to different degrees. As mentioned earlier, antimicrobial therapies are difficult to monitor, so it is very important to use the correct doses of drugs. The dosing schedules of these drugs are used to show the extent to which differences in the pharmacokinetics of the drugs can be caused by the RRT procedure chosen.

### 7.1. Meropenem

Meropenem inhibits bacterial cell wall synthesis in Gram-positive and Gram-negative bacteria by binding to penicillin-binding proteins (PBPs).

The size of the molecule is 437.5 daltons. Notably, 2% of this binds to proteins. The volume of distribution is 0.35 L/kg [36]. These data suggest that the drug can be removed by dialysis, which is supported by the SmPC. The drug needs to be adjusted for renal function (see Table 2).

### 7.2. Vancomycin

Vancomycin is a tricyclic glycopeptide antibiotic that inhibits cell wall synthesis in sensitive bacteria by binding with high affinity to the D-alanyl-D-alanine terminus of cell wall precursors. The drug has a slow bactericidal effect on microorganisms that divide. In addition, it reduces the permeability of the bacterial cell membrane and RNA synthesis.

The size of the molecule is 1449.3 daltons. Notably, 10–50% of this binds to proteins. The volume of distribution is 0.47–1.1 L/kg [36]. These data suggest that the drug is not removable by dialysis, which is supported by the SmPC. The drug needs to be adjusted for renal function (see Table 3).

### 7.3. Imipenem/Cilastatin

Imipenem is a carbapenem-type beta-lactam antibiotic. It acts as an antibacterial agent by inhibiting the synthesis of bacterial cell walls.

Cilastatin sodium is a competitive, reversible, and specific inhibitor of dehydropeptidase-I, the renal enzyme that metabolizes and deactivates imipenem. Cilastatin sodium exhibits no antibacterial activity.

The size of the molecule is imipenem/cilastatin 317.4 and 380.4 daltons. Notably, 20% and 40% binds to proteins. The volume of distribution is 0.23 L/kg and 0.22 L/kg [36]. These data suggest that the drug can be removed by dialysis. This is supported by the SmPC. The drug needs to be adjusted for renal function (see Table 4).

### 7.4. Ceftazidime

Ceftazidime stops bacteria from making cell walls after they attach to penicillin-binding proteins (PBPs). This causes bacterial cell lysis and death by disrupting the biosynthesis of the cell wall (peptidoglycan).

The size of the molecule is ceftazidime 637.7 daltons. An amount of <10% binds to proteins. The volume of distribution is 0.28–0.4 L/kg [36]. These data suggest that the drug can be removed by dialysis. This is supported by the SmPC. The drug needs to be adjusted for renal function (see Table 5).

Additionally, the examples demonstrate that different RRT methods call for varied dose regimens. The method that is employed during RRT therapy determines the rate and effectiveness of drug removal in situations where dialyzing is an option.

## 8. Conclusions and Future Perspectives

In the case of CRRT, dose adjustment of each drug is a recommendation that should be supplemented by the clinical condition of the patient. If possible, the therapy should also be supplemented with therapeutic drug monitoring (TDM). Based on all of these, drug therapy should be planned and developed. Summarizing the above, we can see that we are using a system with many variables. The more complex a system is, the more likely it is to cause effects or side effects at more points.

These side effects may be reversible or irreversible, severe or mild. We can see that, during a CRRT procedure, there are a significant number of independent variables in the system both in terms of indication, timing, length of treatment or drug therapy, which raises questions. It is very difficult to develop a good and accurate therapy from every point of view, but this should always be the goal.

From the point of view of the application of drugs, the different structure and properties of the membranes, the changed physiological conditions, the physicochemical properties characteristic of the active substances, the residual kidney function and the liver function can influence the pharmacokinetics to such an extent that it is difficult to set up a uniform dosage scheme. Renal function replacement achieved with CRRT cannot be regarded as the same as real kidney function of the same value from the point of view of pharmacokinetics. Given how complicated it is to determine the right dose of a drug or the timing of a treatment with CRRT, it becomes understandable why it is strongly recommended to consider the therapy individually for each patient. The above-mentioned basic principles and the technical parameters of the used renal replacement device must be taken into account for the decision.

An important research goal would be to investigate the adsorption properties of drugs that are often used in intensive care. Due to the different composition and properties of the membranes on the market, it would be worthwhile to examine the membranes separately. With the data obtained from these tests, taking into account the adsorption properties of the different membranes, it would be possible to prepare recommendations for dose modification. CRRT is a very versatile, very good procedure in many respects, but we have to realize that many questions still need to be answered in order to know exactly in which cases, when and for how long it is used, as well as how it affects the pharmacokinetics of other drugs used in therapy.

## Figures and Tables

**Table 1:** IHD and CRRT comparison table, showing differences, advantages, and disadvantages.

	IHD	CRRT
Mechanism of removal	Diffusion	Diffusion, convection, or both
Flow rates	Blood flow rate > 200–300 mL/min, Dialysate flow rate > 500 mL/min	Blood flow rate < 200 mL/min, Dialysate flow rate 17–34 mL/min
Duration	3–4 h	24 h/day
Advantages	Rapid, cheap	Hemodynamic stability, better fluid control, to remove medium molecular weight substances

**Table 2:** Meropenem dose adjustments.

Normal renal function	500–1000 mg every 12 h
eGFR 50–26 mL/min	500–2000 mg every 12 h
eGFR 25–10 mL/min	500–1000 mg every 12 h 500 mg every 8 h
eGFR < 10	500 mg every 12 h
**RRT**	
IHD	Dialyzed Dose as in GFR < 10 mL/min or 1000–2000 mg post-dialysis
CVVH/HD	Dialyzed 500–1000 mg every 8 h 1000 mg every 12 h
CVVHDF	Dialyzed 1000 mg every 12 h

**Table 3:** Vancomycin dose adjustments.

Normal renal function	1000–1500 mg every 12 h
eGFR 50–20 mL/min	500–1000 mg every 12–24 h
eGFR 20–10 mL/min	500–1000 mg every 24–48 h
eGFR < 10 mL/min	500–1000 mg every 48–96 h
**RRT**	
IHD	Not dialyzed Dose as in GFR < 10 mL/min.
CVVH/HD	Dialyzed 1000 mg every 48 h
CVVHDF	Dialyzed 1000 mg every 24 h

**Table 4:** Imipenem/Cilastatin dose adjustments.

Normal renal function	500 mg every 6 h
eGFR 90–60 mL/min	400–500 mg every 6 h 750 mg every 8 h
eGFR 60–30 mL/min	300 mg every 6 h 500 mg every 6–8 h
eGFR 30–15 mL/min	200 mg every 6 h 500 mg every 12 h
eGFR < 15 mL/min	200 mg every 6 h 500 mg every 12 h
**RRT**	
IHD	Dialyzed Dose as in GFR < 15 mL/min
CVVH	Dialyzed 250 mg every 6 h 500 mg every 8 h
CVVHD/HDF	Dialyzed 250 mg every 6 h 500 mg every 6–8 h

**Table 5:** Ceftazidime dose adjustments.

Normal renal function	1000–2000 mg every 8–12 h
eGFR 50–31 mL/min	1000–2000 mg every 12 h
eGFR 30–16 mL/min	1000–2000 mg every 24 h
eGFR 15–6 mL/min	500–1000 mg every 24 h
eGFR < 5 mL/min	500–1000 mg every 48 h
**RRT**	
IHD	Dialyzed 500–1000 mg every 48 h or post-dialysis
CVVHD/HDF	Dialyzed 2000 mg every 12 h

## Data Availability

Not applicable.
